# Predictive models for clinical decision making: Deep dives in practical machine learning

**DOI:** 10.1111/joim.13483

**Published:** 2022-04-25

**Authors:** Sandra Eloranta, Magnus Boman

**Affiliations:** ^1^ Division of Clinical Epidemiology Department of Medicine Solna Karolinska Institutet Stockholm Sweden; ^2^ Division of Software and Computer Systems School of Electrical Engineering and Computer Science, KTH Stockholm Sweden; ^3^ Department of Learning, Informatics, Management, and Ethics Karolinska Institutet Stockholm Sweden

**Keywords:** machine learning, precision medicine, clinical decision‐making, artificial intelligence, physician

## Abstract

The deployment of machine learning for tasks relevant to complementing standard of care and advancing tools for precision health has gained much attention in the clinical community, thus meriting further investigations into its broader use. In an introduction to predictive modelling using machine learning, we conducted a review of the recent literature that explains standard taxonomies, terminology and central concepts to a broad clinical readership. Articles aimed at readers with little or no prior experience of commonly used methods or typical workflows were summarised and key references are highlighted. Continual interdisciplinary developments in data science, biostatistics and epidemiology also motivated us to further discuss emerging topics in predictive and data‐driven (hypothesis‐less) analytics with machine learning. Through two methodological deep dives using examples from precision psychiatry and outcome prediction after lymphoma, we highlight how the use of, for example, natural language processing can outperform established clinical risk scores and aid dynamic prediction and adaptive care strategies. Such realistic and detailed examples allow for critical analysis of the importance of new technological advances in artificial intelligence for clinical decision‐making. New clinical decision support systems can assist in prevention and care by leveraging precision medicine.

## Graphical abstract

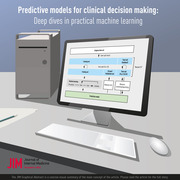

## Introduction

This review focuses on predictive modelling and, in particular, the role of machine learning in precision health. Machine learning covers a broad range of algorithms that aim to automate, and sometimes emulate or imitate, human learning to solve classification or prediction tasks, recognise patterns or identify clusters in data. These models are typically trained and optimised on retrospective data to accurately predict the outcome of future observations that have arisen under the same data‐generating mechanism. The capacity of machine learning to generalise from past behaviour, the traces of which constitute the data, to predict the result of new unlabelled observations fits nicely with the broad purpose of precision medicine, to aid clinicians to provide ‘the right treatment to the right patient at the right time’. A concise definition of precision medicine is *multimodal patient stratification and monitoring*. We stratify into subpopulations, and when good results are achieved, we aggregate back into larger populations. It is estimated that this process is approximately 90% identical for any two diagnoses, and even if the remaining 10% are crucial for precision medicine to work as intended for a particular diagnosis, the similarities are what afford our generalisations. The monitoring component yields massive databases in various modalities, such as text and images related to physiological parameters, which in turn feed the decision‐making processes that govern personalised care and treatment.

Tools that support clinical decision‐making have been enabled through concurrent developments in clinical genetics, bioinformatics, artificial intelligence (AI) and statistics. Research activities are characterised by a high degree of technical specialisation among those who contribute, and therefore prompt collaboration between different experts. This interdisciplinary arena and the clinical–technical collaboration necessary for impactful and actionable results provide the primary motivation for this review. In addition to a shared understanding of the clinical problem to be solved, mixed‐competence teams require a firm grip on key concepts, jargon and the methods used.

Machine learning continues to gain traction in the clinical community; however, the threshold to enter this field can be viewed as high. Our review is intended for clinical researchers and will provide a context for the use of machine learning in predictive modelling, with a particular emphasis on its role in precision health. For readers who wish to study the basics of machine learning, we also aim to bridge the training gap by scoping the medical literature for available tutorials and introductory articles targeted towards clinicians. The purpose of our literature review was to identify articles that may serve as general primers for machine learning methods as opposed to review articles that summarise their application in specific areas of medicine.

We begin by explaining the conceptual differences between estimation models aimed at prediction versus statistical inference. We then continue by describing our review of the introductory literature on machine learning methods for medical research and highlight key references for further reading. Next, we elaborate on the rationale for machine learning in precision medicine research before we finally dive deeper into select topics related to predictive modelling that are not typically covered in introductory texts.

## Prediction versus statistical inference

Data‐driven predictive modelling can be contrasted with statistical modelling approaches used to study hypothesised exposure–outcome relationships with the purpose of understanding or explaining causal mechanisms [1, 2, 3]. The latter has a long history in clinical and epidemiological research and encompasses concepts and methods used in statistical inference, that is, the process of drawing conclusions from data. At its core, methods used for inference, such as hypothesis testing and effect size estimation, seek to separate systematic patterns or differences between groups from random variation. In the observational setting, such efforts often entail ruling out confounding and other forms of bias as possible explanations for any observed statistical associations. Domain‐specific clinical knowledge of the mechanistic or biological processes under study is a key component in the development of statistical models. The classical approach to statistical modelling is both taught and widely used in medicine and is the methodological cornerstone in studies of disease aetiology (risk factor research), treatment efficacy or intervention research, epidemiology and nosology. Statistical inference can, thus, help with identifying and understanding, for example, what patient or disease characteristics act as independent risk determinants for disease relapse or dismal outcomes. However, the model‐building approach to address such research questions is entirely different from the workflow of predictive modelling, as we explain in this article, and the fundamental difference in the objective is important to recognise.

## Artificial intelligence and machine learning

Humans often solve problems by first guessing or estimating a solution, but even if they are intelligent and experienced, they often get it wrong. This is to be expected; we often hear that such mistakes make us human. When machines ‘guesstimate’, algorithms calculate the nature of their error mathematically or logically and attempt to minimise it over many superhumanly fast iterations. For simple and linear problems, such error correction works well and optimal solutions are quickly reached. If the problem is complex and non‐linear, it is usually beyond human reach, but algorithms might be able to efficiently calculate a solution in many instances of interest.

In AI, algorithms that search for the best ways to inductively jump to conclusions and then automatically correct for errors have been devised for 70 years. Significant progress has been made in both theory and practice. Of special interest are models that learn how to best solve problems over time and over tasks. In such machine learning, learned structures are retained, what Alan Turing called ‘Indexes of Experience’, and reused. The model, faced with unseen examples, can refer back to the appropriate index and recognise the problem as belonging to a family, and then reuse a solution successfully used for a different member of that same family. Machine learning models thus learn rules (or concepts) from examples, even if they are never fed any rules as such. The rules emerge in sometimes surprising and impressive ways, even to their human programmers. The classic definition by Mitchell captures this [4, p. 2]: *A computer program is said to learn from experience E with respect to some class of tasks T and performance measure P, if its performance at tasks in T, as measured by P, improves with experience E*. Self‐learning and active learning are ways of learning that, over time, become even more useful. Thus, we can say that AI is the field of building autonomous learning systems, whereas machine learning is a part of that field, focusing on models of learning. The models used in machine learning are often further subdivided. For pattern matching and simpler prediction tasks, supervised learning using data annotated by humans can be used. Unsupervised learning can be used for classification and more exploratory means to understand data. Finally, for situated learning agents, a simple mechanism of punishment and reward was used in the process called *reinforcement learning*.

## Introduction to machine learning for clinicians: a targeted literature review

With the arrival of machine learning in medical and clinical research, there is a steadily growing number of articles that aim to serve as introductions to the methodology used. We reviewed the literature for articles that targeted a broad clinical readership and summarised the main focus areas covered in the identified references. The general introduction provided by these articles was used as a steppingstone to our deep dives in the next section.

## Eligibility, screening and selection

We limited our search to PubMed, which is the database of choice for clinicians and researchers in medicine. Articles were eligible if they were published within the last 5 years (October 2016 or later) and written in English. A combination of MeSH terms and title/abstract keywords was used to define the search query (see the Supporting Information for details). The initial search generated 3439 titles that were screened for eligibility. We were interested in identifying non‐specialist primers, topic reviews, tutorials and perspective articles written for a general clinical audience. Therefore, in the screening process, we excluded titles that, for example, indicated that the main purpose of the article was to review or discuss:

- the ‘machine learning hype’ and how AI might transform or ‘revolutionise’ (*sic*) medicine or deliver changes to the clinical setting in the future;
- the use of machine learning in drug discovery, drug development, pharmacovigilance or clinical trial design;
- approaches to explainable AI or topics related to ethical AI;
- comparisons of predictive or prognostic models in specific research areas;
- theoretical aspects of machine learning and AI written for methodology specialists.

**Fig. 1:**
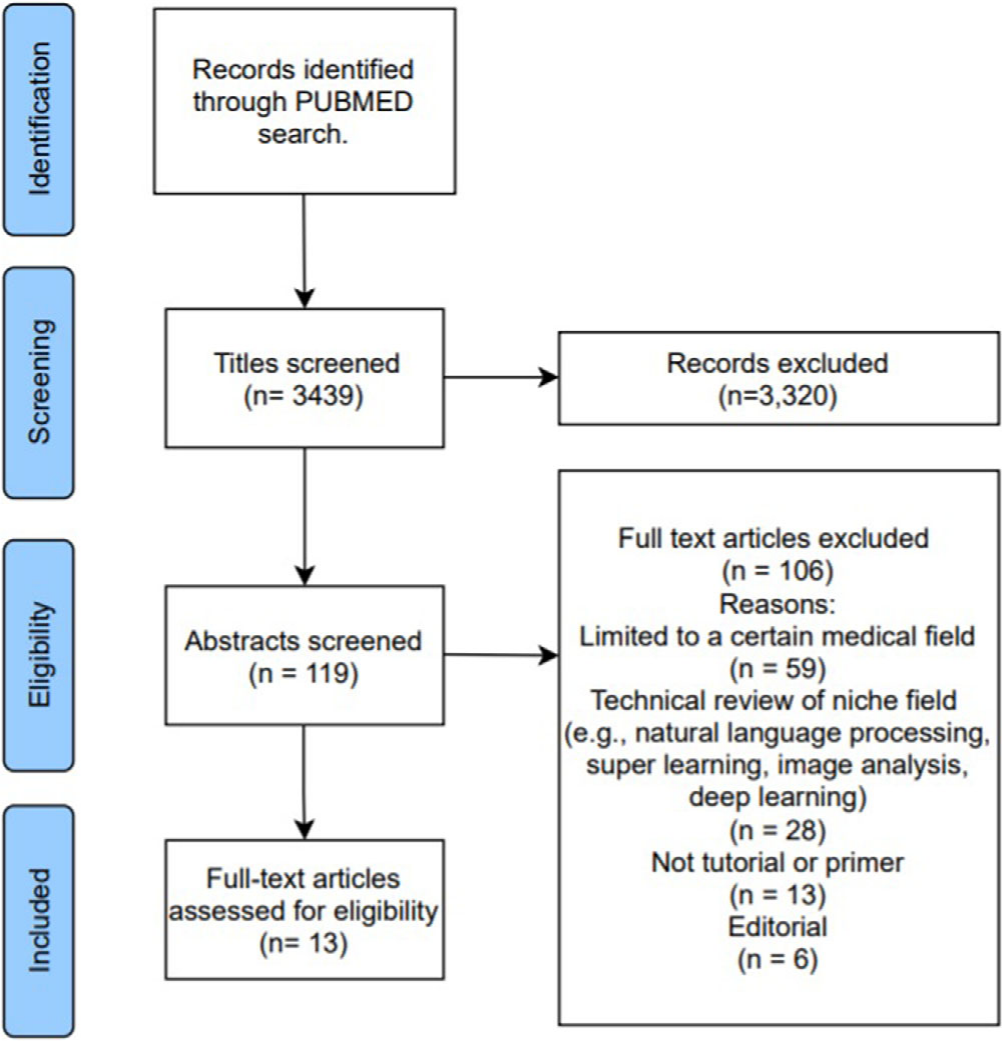
Prisma flow diagram for the article selection process.

The screening process resulted in 119 articles that were eligible for abstract review (Fig. 1). Among these, we excluded articles that summarised the clinical applications of machine learning in specific fields (e.g. ophthalmology, psychiatry, dentistry, oncology and cardiovascular disease), and articles that reviewed machine learning methods used specifically in niche areas, such as natural language processing or deep learning methods used in radiology. Of the 13 articles that remained and were read in full, five were further excluded as they were either not introductory review/tutorial articles (*n* = 3) or targeted towards a specific medical field (*n* = 2).

## Key introductory articles for clinicians

Table 1 summarises the overall aim, choice of machine learning methods explained and any special topics given extra focus in the selected articles. The shared objective of the listed references was chiefly to equip the reader with the terminology used in machine learning and to explain some of the broad taxonomies (e.g. supervised, unsupervised and reinforcement learning) and key topics relevant for designing or evaluating machine learning projects. Some of the central concepts in the prediction methodology include the bias–variance trade‐off, model performance metrics and pipelines for model development with their corresponding workflows. These ideas were elaborated on to some extent in all articles, with particular emphasis on practical challenges and possible solutions in some [5, 6]. Explanations of specific algorithms used in machine learning have focused primarily on methods used in supervised learning (prediction methods for classification or regression tasks that assume that data are labelled, i.e. there is an annotated outcome variable in addition to the set of predictors). Classical statistical methods such as logistic and linear regression (with or without regularisation) as well as machine learning algorithms, including support vector machines and classification and regression trees (and ensemble variants such as random forests), were covered most often, followed by naïve Bayes classifiers, *k*‐nearest neighbour and the general idea behind artificial neural networks. The main tutorial elements in the selected papers included a guided approach for designing a machine learning project [5] and a hands‐on introduction to implementing machine learning algorithms in R statistical software [7].

**Table 1:** Articles identified in the literature review that introduce machine learning to clinical readers

Author, title, year and journal	Aim	Methods or algorithms explained	Highlighted topics
Scott, I. A. *Demystifying machine learning ‐ A primer for physicians* 2021 (Intern Med J)	To provide a non‐technical introduction to central concepts in machine learning. The review is structured as a stepped guide for developing and evaluating machine learning models. More advanced topics are supplemented with Web links to video tutorials.	**Supervised learning**: Support vector machines, Naïve Bayes classifiers, K‐nearest neighbour, decision trees, regression models (linear and logistic), artificial neural networks (convolutional neural networks, recurrent neural networks, generative adversarial networks) **Unsupervised learning** *K*‐means clustering, principal component analysis.	Includes a summary of various performance metrics (including evaluation metrics for classification, regression and calibration). Discussion of issues related to deployment, for example model generalisability, explainability, utility and safety.
Shamout F, et al. *Machine learning for clinical outcome prediction* 2021 (IEEE Rev Biomed Eng)	To provide a patient‐centric perspective on learning models, explaining time series and other means to dynamically modelling patient trajectories. Access to data e.g. in EHRs and how to best share data is also discussed. A relatively technical introduction to outcome prediction is also presented.	**Supervised learning**: Support vector machines, artificial neural networks	The paper is laid out in sections that describes an overall machine learning pipeline, with examples of methods and metrics used in each step of the workflow. Highlighted topics include a range of techniques for obtaining embeddings (as a means for dimensionality reduction), abnormality detection and a discussion of model interpretability. So‐called self‐supervised learning (supervised learning without labels) is also explained, via autoencoders. The latter are intelligent compression algorithms that transform high‐dimensional input into near perfect low‐dimensional output of the same thing.
Jiang, T. et al. *Supervised machine learning: A brief primer* 2020 (Behav Ther)	To review topics relevant for prediction tasks, with focus on methods for supervised learning, approaches to model building, evaluation and validation. Also provides a glossary for terms that are used interchangeably in the statistics and machine learning literature, respectively	**Supervised learning**: Random forests, support vector machines	Includes a discussion on approaches to modelling (description, prediction and causal inference) in the context of the research question. Covers some more advanced topics, that is super learning (stacking and ensemble models), regularisation and challenges with external validation.
Maleki F. et al. *Overview of machine learning Part 1: Fundamentals and classic approaches* 2020 (Neuroimaging Clin N Am)	To explain the basic workings of common algorithms used in machine learning (both supervised and unsupervised) and to outline standard workflows. This article is the first part in a two‐part article series by the same authors. The second review article gives an introduction to deep learning methods used in medical image analysis.	**Supervised learning**: Support vector machines, *K*‐nearest neighbour, Naïve Bayes classifiers, decision trees (and random forests), artificial neural networks **Unsupervised learning** *K*‐means clustering, hierarchical clustering	A substantial part of the paper is devoted to explaining the machine learning model development workflow and associated terminology and concepts (e.g. steps in data preparations, bias‐variance trade‐off and performance evaluation metrics).
Lo Vercio, L. et al. *Supervised machine learning tools: A tutorial for clinicians* 2020 (J Neural Eng)	To provide a solid introduction to supervised learning that includes motivation for using it in medical research, review key concepts and present practical advice for a generic workflow. The paper also includes a tutorial part that guide researchers who seek to design a machine learning project.	**Supervised learning**: *K*‐nearest neighbour, support vector machines, Naïve Bayes classifiers, decision trees (and random forests), artificial neural networks (including convolutional neural networks)	This paper includes several sections that share advice for best practice when structuring a machine learning project. A large section of the paper is devoted to assessment of model performance and to explaining a range of performance evaluation metrics.
Badillo, S. et al. *An introduction to machine learning* 2020 (Clin Pharmacol Ther)	To provide an introduction to a broad range of general concepts in machine learning, and to equip readers with tools that can support them to understand research that employs machine learning. The review provides key take‐home messages targeted chiefly towards researchers in clinical pharmacology	**Supervised learning**: *K*‐nearest neighbour, Naïve Bayes classifiers, decision trees (random forests), support vector machines, artificial neural networks (recurrent neural networks, long short‐term memory networks, gated recurrent networks) **Unsupervised learning** *K*‐means clustering, density clustering, hierarchical clustering	Highlighted topics relate to model selection strategies, indicators for model complexity and goodness of fit.
Sidey‐Gibbons, J. A. M. et al. *Machine learning in medicine: A practical introduction* 2019 (BMC Med Res Methodol)	To give a hands‐on introduction to applying machine learning algorithms in the R statistical software. Using a publicly available breast cancer data set, (i) a regularised logistic regression model, (ii) a support vector machine and (iii) an artificial neural network are detailed and evaluated. R code for analyses, performance evaluation and generation of figures is provided.	**Supervised learning** Support vector machines, single‐layer artificial neural networks	In addition to the practical introduction to supervised machine learning, the presentation in this paper discusses and demonstrates ensemble learning as well as natural language processing for analysing linguistic data.
Handelman, G. S. et al. *eDoctor: Machine learning and the future of medicine* 2018 (J Intern Med)	To explain core concepts and common algorithms used in machine learning. The aim is to familiarise clinicians with common methods and metrics that will enable them to understand and evaluate research articles that use machine learning	**Supervised learning**: Support vector machines, artificial neural networks, decision trees (and random forests) **Unsupervised learning** *K*‐means clustering	The first half of the paper has a machine learning basics focus, while the second holds methodological advice as well as examples of applications in precision medicine, therapeutics, radiology, haematology, oncology and pathology. Semisupervised learning is also explained briefly.

We opted to exclude articles organised as checklists for reporting [8, 9], or tools for reading and evaluating the scientific quality of machine‐learning applications [10, 11, 12]. Nevertheless, some of these articles may be of interest to clinical researchers. We also recognise that many additional useful articles would have particular relevance for specialists in specific medical fields, such as rheumatology [13], haematology [14], cardiovascular medicine [15] and psychology [16].

## Machine learning in practice

Supervised learning relies on labels. Nowadays, human experts are more frequently asked to label data items in a binary or categorical fashion. Such requests are motivated by data‐driven AI to allow for automated analysis and learning. Supervised and, in particular, deep machine learning uses validation via quantitative comparison. A 100% accuracy on a classification task means the algorithm gets it correct every time, and *correct* is defined by a human panel of annotators because the quantitative comparison measures the amount to which the computer can emulate the annotations. The latter is often referred to as the *gold standard*. In a prediction task, algorithms are used to beat baseline performance, which is (unlike traditional statistical reasoning) typically not defined as random predictions. Instead, panels of human experts might perform at a state‐of‐the‐art level: a performance level that the learning algorithms then seek to improve upon, either on their own or by merging human predictions with machine predictions. In either case, the clever human programming of learning algorithms provides these algorithms with AI.

Machine learning can handle multiple outcomes. A set of input variables can be used to predict not only the primary outcome but also the secondary outcome. However, a model can also predict a vector of outcomes (i.e. more than one primary outcome) at the same time. Because the outcomes may be interrelated in such cases, a model that considers the dependence between the outcomes is preferable. Simply slicing a prediction task into *n* models, where *n* is the number of elements in the outcome vector, will typically not suffice. For multiple‐outcome predictions, only certain families of learning models are appropriate. Decision tree algorithms work well, for instance. Such algorithms work by cleverly and repeatedly dividing the feature space, with the random forest being a popular choice because of its generality and efficiency. They can be considered as following a path through a tree where, at each split in the tree, the choice about which branch to follow next is made in favour of the best average of the *n* outcomes. Unlike ordinary regression, when our only option is to look at all combinations of *n* (binary or categorical) outcomes, the decision tree methods scale well.

Fair validation of machine learning models in clinical contexts, that is, how advanced models perform in the real world and not in some sandbox, is less straightforward than one might think. In an oft‐cited example of pneumonia detection from labelled frontal chest X‐ray images [17], machine learning algorithms outperformed a panel of four radiologists. Accuracy was measured as the area under the curve (AUC; 0.76 in the case of the algorithm), and the conclusion was that the algorithm outperformed the human panel. However, radiologists could and would diagnose much more than pneumonia in any given scan (cf. [18]) and, therefore, an ensemble of algorithms could and arguably should be compared with a single radiologist. Alternatively, such an ensemble could through so‐called transfer learning, that is, moving between diagnoses and patient populations on the strength of having a general semantic knowledge representation capacity, be compared to a human panel of specialists in diverse fields, all related to the data at hand in one way or another. There is also the long‐term usefulness of stable and efficient algorithms to consider: what if the algorithms keep improving their performance as the number of examples they are exposed to increases? For example, if another million X‐ray images are used for batch (offline) training, the area under the curve may increase. This is often detectable in practice as a new version of a software package. Analogously, short‐term can mean that results such as AUC = 0.76 are contingent on a particular sample (*n* = 112,120 in the cited study) or that they were obtained under harsh budget constraints.

**Fig. 2:**
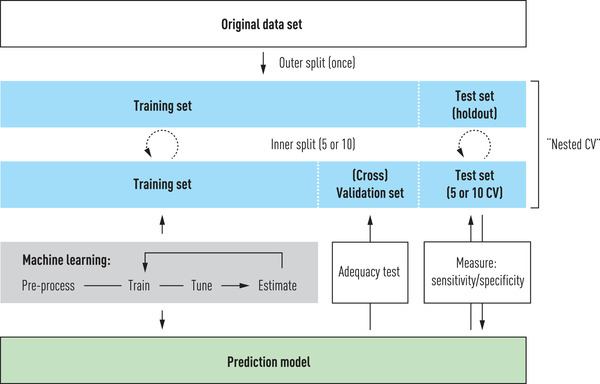
Example workflow for training, testing and validating prediction models.

It is common for modellers to not have domain expertise, but this is not always a drawback. Agnostic modelling and domain experience sometimes meet halfway, the former informing clinicians by showing what is possible to predict, and the latter directing the efforts of the modellers towards targets that are useful in practice. The greatest challenge in domain adaptation is validation. Machine learning systems are fed retrospective training data in a *training* phase (Fig. 2). Some form of pre‐processing is always necessary, as the original dataset will not be free from errors (such as duplicate or missing entries, integrity constraint violations or outlier values), and will need to be represented in a data frame (a matrix) on which the algorithms can operate. After training and performing what is often called hyperparameter tuning, that is, relaxing point values to intervals, mainly to provide more freedom to the algorithm to combine values in a very high‐dimensional representation, their prediction capability is then estimated. The results are then further examined in a *testing* phase. Testing is often performed on data identical to the training data or a subsample, but a proper holdout can also be used. At the very least, test data are similar to what the system has seen previously and should therefore not prove difficult to predict or classify from. Therefore, it is common to observe near‐perfect accuracy in tasks during the testing phase. Naturally, such perfect learning is overfitted to the training data and, as such, is a poor indicator of how the system would perform on previously unseen data [19]. This motivates a *validation* phase, where the system takes as input a holdout sample or data that did not even exist during testing. During validation, we can assess the prediction model for real, which often prompts going back to more training and testing. It is imperative that the workflow does not allow cherry‐picking, but instead stress tests the results to the maximum [20]. The static illustration in Fig. 2 captures neither the dynamicity of new data arriving nor the saving and reuse of learned structures that are sometimes possible. However, it captures the basic structures for validating a prediction model, which can then be measured in terms of the receiver operating characteristic (ROC)‐AUC, F1, balanced accuracy or some other quantitative score to be compared with a baseline or benchmark. In addition to holdout sampling with a dedicated subsample reserved for taking the role of completely unseen data, a 5‐ or 10‐fold cross‐validation is usually carried out, in which 20% or 10%, respectively, is selected at random. Random seeds for any stochastic variable are kept for replication purposes, and any particulars of the programming platforms (e.g. TensorFlow or PyTorch) used are also noted. Most importantly, the code is shared in full, and if data are too sensitive to include, synthetic data or pseudonymised examples should at least be included.

With all these questions surrounding the validation of machine learning algorithms, it is tempting to let domain experts decide what provides clinically actionable insight. Algorithms are agnostic until they are given domain knowledge to represent and reason. We argue that such knowledge could be presented to the learning algorithms in both correct and incorrect ways, and that this, in turn, dictates what the algorithms could provide in terms of optimal performance. As clinicians often invoke risk scores by which patients are binned into groups that need immediate attention, monitoring etc., such at‐risk labelling entails binning. For a clinician, a risk score value is not a simplification as much as it is an aid to attend to the patient correctly, not least because it may be part of a standardised clinical workup routine or treatment guidelines. However, dichotomisation means discarding potentially important information and, as we will see, it can make an algorithm learn in a suboptimal manner. The prediction capacity of the clinician is incredibly high because of the rapid and efficient adaptation to context and pragmatics of care. In contrast, computers deal with procedural (algorithmic) and declarative (logical) representations, caring little for pragmatics. A concrete example of a complex disease and its treatment will illustrate how and why this is important.

### Deep dive I: Precision psychiatry

**Fig. 3:**
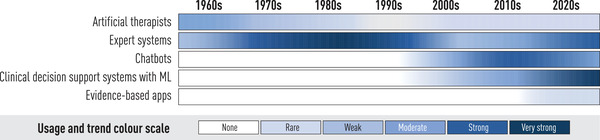
Trends in development of AI systems within psychology.

Psychology and AI have a long history, with the trends indicated in Fig. 3. Human decision‐makers often dichotomise continuous values into discrete risk scores. An example is the Montgomery–Åsberg Depression Rating Scale (MADRS) for major depressive disorder (MDD) [21]. In earlier work, based on patient self‐reporting, we showed how machine learning may help assess which MDD patients in Internet‐based treatment should receive extra attention from therapists [22]. This is relevant to precision medicine because it allows for a move from stepped care to adaptive care by providing dynamic decision support to psychologists. We have previously shown by statistical reasoning without machine learning that empirically derived clinician preferences for actionable predictions were obtained from relatively simple and limited data (in the form of weekly reports), albeit fairly late, almost halfway through the 12‐week treatment for MDD [23]. If predictions of who might fail to benefit from the programme could successfully be made earlier, say at the end of week 3, interventions could also follow before the patient loses interest in, or the ability needed for, remaining active on the treatment platform.

As a rudimentary baseline, one may choose random predictions, but more often, human activity determines performance metric values to improve upon. In the classical statistical model mentioned, treatment weeks were the smallest unit of time for such metrics, and for MDD, chance (defined as the lower bound of the 95% confidence intervals exceeding 50%) was beaten before treatment started using only screening data. The clinically derived baseline was 65%, motivated by descriptive rather than prescriptive argumentation [24], which improved monotonously from week 6. We then investigated how much we could improve upon the time to intervention with the help of natural language processing. If successful, such an approach would also eliminate the need for manual work by therapists to realise an adaptive strategy, replacing the expert heuristics used in the statistical work by automated data analyses. In a relatively simple analysis using four different word‐embedding methods, we used retrospective data (180,017 answers from homework reports and 146,398 direct messages), totalling almost 29 million linguistic tokens [25]. Our goal was to predict who were to become neither responders (at least 50% symptom reduction, using MADRS‐S and a cut‐off widely used by clinicians [26]) or remitters (again using an accepted cut‐off of 10 for an interval [0, 54] of possible MADRS‐S values). Our results indicate that there is a signal: even individual trajectories through the treatment programme with a proxy as simple as concatenated answers to homework reports have predictive power. The signal was rather weak; however, only one of the four models beat the clinically derived baseline in a shorter time than the statistical model and not by much. If the difference were considerable, we would have considered optimising the model via hyperparameter tuning.

Trust in a model can only come from its grounding in the clinical setting. Part of the model grounding comes from predicting the outcomes that the clinicians are interested in, rather than letting model tuning illustrate everything that can theoretically be predicted. In this case, in addition to the primary outcome of those at risk of not benefitting from treatment, the secondary outcomes were as follows:

- responder;
- remitter;
- clinical significant change;
- deteriorated;
- non‐responder.

Note that these outcomes do not partition the set of all possible outcomes. For instance, it is not obvious why non‐responders were included, but not non‐remitters. In short, being a remitter but a non‐responder is positively associated with low initial severity, as observed empirically and affirmed by modelling [23]. Second, they all pertain to points in time and can be modelled as a time series. For example, deterioration compares pre‐ and post‐treatment values using the reliable change index, which is a measure of clinically significant change. Referring back to the first note, in the case of deterioration, there is an overlap with non‐responders.

Rather than tuning our simple natural language processing model, we are currently considering a so‐called transformer, a powerful deep learning model [27], for analysing free text messages sent from patients to therapists. A transformer uses a high‐dimensional structure for its contextualised semantic analysis of natural language, and may have general‐purpose language understanding skills enhanced via additional training on domain‐specific text. It is a contextual model (i.e. resting on the assumption that ‘you shall know a word by the company it keeps’ [28]) of a kind that computational linguists have used for decades and that rests on lexical co‐occurrence in word embeddings. Training of a transformer can be performed by taking the entire Wikipedia in English and blanking out words that the transformer would predict. This is an efficient strategy because it requires no labelling at all, and the gold standard consists of blanked out words in their respective contexts. This is how contextualised semantic models slowly take form. Once trained on massive amounts of text at a great energy expense, a transformer can be used for a vast range of language representation and understanding tasks. For domain‐adapted deep learning, for which the system will train on real data from the treatment programme, labels for primary and secondary outcomes can be used to allow testing and validation.

A trade‐off becomes apparent at this stage, in which the clinically relevant outcomes describe an ideal: perfect accuracy is achieved by meeting the gold standard. This occurs when the labels applied to retrospective data, for which the true outcomes are known, can be perfectly predicted or classified by the transformer. However, as we have seen, the outcomes considered relevant involved descriptive threshold values and MADRS‐S scores. None of these will be useful to the machine learning algorithm in their own right; they were developed by humans for human use. The treatment programme was dichotomised into weeks. If a clinically significant change is calculated to hold on a Monday or Friday of a certain week, it will not be significant to the outcomes, as calculated by the humans responsible for the treatment programme. For the algorithm, this becomes problematic because what it is supposed to learn in training is not precisely when something is predicted to happen but in which week. Analogously, the algorithm learns to recognise a responder based on training examples where each person appearing is labelled either as a responder or non‐responder. This is different from training for estimating a precise score for that person. In quantitative terms, the difference between two people labelled differently can be much smaller than that within the two categories. Unlike human decision‐makers, the algorithm does not appreciate the value of handles or simplifications; it simply computes. Hence, the trade‐off is between letting the algorithm learn from the human annotations, with their simplifications, or from discarding the dichotomisations entirely, avoiding simplifications and using all the data points. In the latter case, the potentially optimal quantitative results must somehow be translated back to clinically actionable insights using the terms and outcomes of human decision‐makers. For each domain, arguably even for each treatment, this is a delicate and non‐trivial task. Many researchers in machine learning call for transparency, arguing that optimal results may be worth giving up on whether algorithms remain intelligible. Naturally, this argument only holds if the less than optimal results are intelligible to human domain experts, which is not a given: any machine learning system takes some effort to grasp, even for people used to data‐driven reasoning.

### Deep dive II: Predicting survival outcomes after aggressive lymphoma

Aggressive lymphomas form a heterogeneous group of malignancies that are highly chemotherapy‐sensitive but where 20%–40% (depending on subtype) of patients still encounter a relapse. Early relapses, or relapses in the central nervous system (CNS), are associated with a particularly dismal prognosis, and understanding how new molecular biomarkers can support clinical decision‐making and individual treatment adaptations in high‐risk patients will be central to precision medicine in lymphoma [29].

Survival analysis is used extensively in these efforts because of its ability to handle time appropriately. A feature of cohort studies, a common study design for prognostic or predictive modelling, is that the exact time points at which patients enter and leave the cohort may vary between individuals due to censoring. Censoring occurs in patients for whom follow‐up is only partially observed, for example, when restrictions are applied to the observation window or when patients are lost to follow‐up [30]. Classical survival analysis often handles differential follow‐up times by estimating *hazard rates*, which can subsequently be converted and expressed in terms of survival probabilities via the one‐to‐one relationship between the two metrics [30]. Despite the frequent use of cohort studies in medical research, machine learning algorithms adapted for survival data have rarely been mentioned in introductory texts. Nonetheless, there is a broad range of methods that incorporate follow‐up time, which can be used for classification problems with survival data [31]. These include both traditional statistical methods (e.g. conventional or regularised variants of Cox regression, accelerated failure time models and various parametric models) [32] and machine learning algorithms that have been adapted for censored data (e.g. random survival forests, support vector machines, Bayesian methods and neural networks) [33]. Evaluation metrics for the performance of survival prediction models (e.g. time‐varying AUCs, Youden's index, Brier scores, Harrell's C‐index) have also been developed and widely used in practice [33, 34].

In the current clinical routine for lymphoma management, variants of the International Prognostic Index (IPI) scores that build on the patient's age, performance status, lymphoma stage, extra‐nodal location and serum lactate dehydrogenase level are used to form risk groups (low, low–intermediate, intermediate–high or high risk) that, in turn, inform treatment decisions. For diffuse large B‐cell lymphoma (DLBCL), the standard of care for patients deemed fit for curative treatment is the immunochemotherapy regimen R‐CHOP (the monoclonal antibody rituximab plus cyclophosphamide, doxorubicin, vincristine and prednisolone). Among patients selected for this treatment, the most critical period in terms of the prognostic outlook is the first 2 years after diagnosis, during which time close to 25% of patients are anticipated to be refractory to first‐line treatment or relapse, with approximately 4% of patients encountering a relapse in the CNS [35]. The clinical challenge is to identify high‐risk patients early who might be eligible for new targeted biological treatments in combination with standard primary chemotherapy or other individually adapted treatment schemes in the future. Improved treatment tailoring in relapse settings is highly warranted. In an attempt to challenge the discriminative capacity of the IPI, a new prediction model for overall survival was recently developed using an ensemble approach that stacked 14 different survival models to improve predictive accuracy [36]. The training of the models was performed on Danish population‐based data that included up to 34 host and tumour factors, which made different assumptions on the parametric form of the baseline hazard, functional form of continuous variables, time‐dependent effects and interaction effects. Swedish population‐based data were used for validation. The stacked model (implemented at https://lymphomapredictor.org), with a C‐index of 0.744 in the Swedish data, outperformed the IPI (C‐index of 0.661), as well as all other individual models that were weighed into the ensemble. The time‐varying AUC further indicated that the stacked model performed consistently better than the IPI across all points of follow‐up (up to 5 years after diagnosis).

Similar to the MADRS‐S score discussed previously, the IPI uses dichotomised representations of the included variables when summing up the scores for individual patients. For example, in DLBCL, points are assigned for age >60 years, stage *>*3, elevated serum lactate dehydrogenase, performance status *>*2 and >1 extranodal site. This dichotomisation, in particular of age which is a very strong prognostic factor, leads to substantially lower predictive accuracy of overall survival in patients with DLBCL [37]. Comparable differences in predictive performance, as evaluated by the time‐varying AUC, have also been demonstrated for seven other forms of lymphoma (including advanced Hodgkin lymphoma, follicular lymphoma and mantle cell lymphoma) when comparing Cox regression models that included a continuous representation of the effect of age and a non‐categorised version of performance status to established subtype‐specific variants of the IPI (e.g. FLIPI for follicular lymphoma and MIPI for mantle cell lymphoma) [38].

In the above examples, the main gain in terms of model performance was attributable to the more efficient use of the data (i.e. by not discarding information). This was achieved using relatively parsimonious classical statistical models that included few, but well‐known, clinical predictors of survival. When a cross‐validation approach for selecting the best model among a set of possible models was instead used (cf. [39]), and where machine learning algorithms, such as random survival forests and Cox regression with a ridge penalty, were also considered, the benefit in terms of predictive performance was essentially the same as for the classical statistical models [38]. This is not to say that machine learning is superfluous in lymphoma prediction, but more likely just reflects the relatively simple prediction task for which modelling of non‐proportional and non‐linear effects, as well as interactions in the conventional survival models, was still practically feasible. The strength of machine learning becomes clear in more complex prediction settings, for example, when the data are multimodal, unstructured (e.g. in free‐text fields in an electronic health record) and/or high‐dimensional [40], scenarios in which classical statistical methods are likely to either perform sub‐par or fail altogether [41].

Looking beyond the methodological intricacies of prediction that aims to identify high‐risk patients, lymphoma is inherently genetically diverse. In DLBCL, the clinical utility of subtype classifications based on cell of origin (germinal centre B‐cell like [GCB], activated B‐cell‐like [ABC], unclassified DLBCL) or, more recently, on molecular features that require comprehensive genetic sequencing, is still only suggestive [42, 43, 44]. For example, the extent to which sequencing of tumour tissue at diagnosis will contribute to more detailed lymphoma subtype classification and identification of predictive markers that can guide the choice of treatment in the first‐ or second‐line remains at the forefront of lymphoma precision medicine research. Harkins et al. neatly summarised the following:

Successful integration of predictive and prognostic tools in clinical trials and in a standard clinical workflow for DLBCL will likely require a combination of methods incorporating clinical, sociodemographic, and molecular factors with the aid of machine learning and high‐dimensional data analysis. [45, p. 959]

**Fig. 4:**
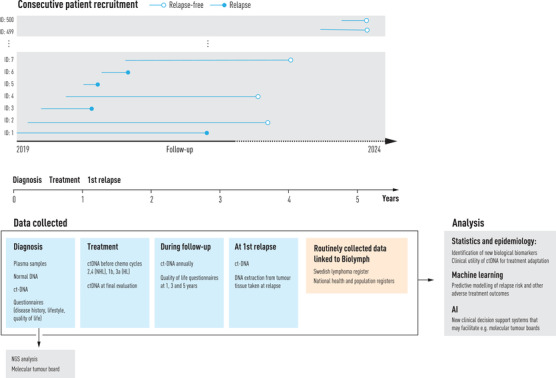
Overview of patient recruitment and data collection in the BioLymph study.

To this end, Biolymph, a prospective study that acts as both a clinical development project and a research project, with the broad aim of improving diagnostics, treatment and follow‐up for lymphoma patients, was rolled out at the Karolinska University Hospital in Stockholm, Sweden, in early 2019. Patients are enrolled consecutively with tumour tissue and plasma samples sent for genetic analyses at diagnosis (Fig. 4).

This study uses a validated broad next‐generation sequencing panel (GMS lymphoid) that has been developed specifically for lymphoma and covers 252 genes. Analyses of liquid biopsies, or cell‐free tumour DNA (ct‐DNA), during and after lymphoma treatment are further carried out to investigate whether and how individual liquid biopsy profiles can improve the assessment of first‐line treatment response and complement or replace current radiographic evaluation methods. The hope is that the Biolymph study will contribute to practice changing non‐invasive means for actively monitoring treatment response that might help to identify high‐risk patients early and to develop and implement individually tailored treatment schemes and follow‐up routines in lymphoma. To further promote rapid clinical uptake and implementation of important findings, molecular tumour boards for multidisciplinary expert panel discussions on individual results are used in the study. In terms of analytics in Biolymph, the prediction tasks will be more complex and likely require joint modelling of longitudinal and survival data, simultaneous modelling of competing risks (e.g. the risk of death when predicting the risk for relapse) using a mixture of data types such as demographic and clinical data from administrative registers, patient‐reported outcomes, genetic and other biomarker data (e.g. ct‐DNA profiles) from laboratories and data abstracted from medical records. For this purpose, machine learning will undoubtedly be an important tool for further understanding the potential clinical utility of new predictive markers for lymphoma.

## Discussion

**Fig. 5:**
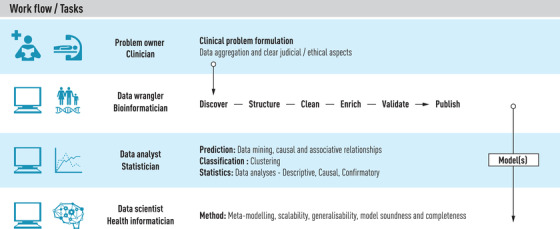
Overview of roles and tasks in predictive modelling.

This review took as a starting point a summary of non‐technical introductory tutorial articles that can help approach the main ideas and principal workflows used in predictive modelling, some technical jargon and a few methods commonly used in machine learning. However, as the growing literature on machine learning and AI applications in medical research suggests, this field is no longer considered a novelty. In contrast, the future potential for the implementation of precision medicine in clinical care, whether for prevention, diagnostics or intervention, has set new demands on collaboration between multidisciplinary teams, both in research and in the clinic. There are many and varied roles that must be filled to develop a useful and sound predictive model. In Fig. 5, we indicate such roles for four consecutive sets of tasks, with the data‐driven role indicated first, followed by the healthcare role. An important exception is the top task, for which the role of the Problem owner could be equal to that of the clinician or at any rate should be chosen among the domain experts. Machine learning proper, as detailed in Fig. 2, starts with the third set of tasks in the workflow and continues through the fourth.

Any clinical decision‐making system based on AI leaves traces in the form of data as well as metadata on how the data were processed. The metadata can include the adequacy and use of the results computed. It is quite common to observe many different machine learning methods employed in such systems. If privacy concerns make a system difficult to implement, it can be difficult to maintain over time. If consent and GDPR issues were solved for a randomised controlled trial, and this trial proved successful and the system was CE marked, for how long can the system be considered the same system [46]? Because it is learning, does the system have to be reinvestigated for CE marking, for instance? Because any AI system uses methods that learn from data, can all data or only some data be kept, perhaps metadata or pseudonymised data? These are examples of practical issues and challenges that require regulatory oversight and IT solutions to facilitate uptake.

The reality of clinical research is typically more complex than what can be covered in any review article, as exemplified through a question posed to us by a clinician during writing: if we have a set of data defining clinical phenotypes and we have multiple different known outcomes, can machine learning analyse the data to sort out which phenotype predicts which outcome? Thus, as we have discussed, the answer is yes, but we still need to consider various properties of the set of outcomes and select the right learning method. If the outcomes are very similar or very different from each other, a machine learning model might struggle when computing averages to divide the feature space efficiently. Deep learning can achieve this, owing to the hidden layers that allow them to learn a non‐linear mapping from the original input variables (phenotypes, in this case) to the ideal features for learning multiple outcomes. However, they are costly to train, and it might be difficult for clinicians to precisely understand the contribution of each phenotype to each of the outcomes. A decision tree model is then a more pedagogic choice, and for random forests, for example, there are many tools for visualising feature importance.

For complex disorders, such as those studied and treated in psychiatry, realistic goals for using machine learning at the clinic must be set. For treatment adherence and activity, adaptive care for individuals, and for assisting classical epidemiological studies on retrospective data, there is much to test and validate, and much of the data needed are already available. A likely way forward in lymphoma involves the development of disease‐specific clinical decision support systems to facilitate, for example, molecular tumour board meetings, where individualised patient management plans are shaped with input from a multidisciplinary team of healthcare professionals. This will call for efficient approaches for comprehensive summaries of the patients’ full clinical picture (molecular, clinical, demographic etc.), evaluation of plausible disease trajectories, and weighing of new research developments and results.

## Author Contributions

Sandra Eloranta: Conceptualization; Investigation; Methodology; Writing – original draft; Writing – review & editing. Magnus Boman: Conceptualization; Investigation; Methodology; Writing – original draft; Writing – review & editing.

## Conflicts of interest

The authors report no conflicts of interest.

## Supporting information

Supporting information
