# Supplementary material for: Predictive models for clinical decision making: Deep dives in practical machine learning
Source: J Intern Med. 2022 Apr 25;292(2):278–95. doi: 10.1111/joim.13483 (PMC9544754; doi:10.1111/joim.13483)
Supplement: Supplementary file 1 — Supporting information [file JOIM-292-278-s001.docx]

**Supplementary material**

**PubMed search query**

The following set of MeSH terms and keywords were used to identify articles eligible for our scopoing review. The search was conducted in PubMed on October 2021.

(("Machine Learning"[MeSH Terms] AND ("Physicians"[MeSH Terms:noexp] OR "Clinical Decision-Making"[MeSH Terms] OR "decision making, computer assisted"[MeSH Terms:noexp])) OR ("machine learning*"[Title/Abstract] AND ("introduct*"[Title/Abstract] OR "primer"[Title/Abstract] OR

"tutorial*"[Title/Abstract] OR "clinical setting*"[Title/Abstract] OR

"clinician*"[Title/Abstract] OR "medical professionals"[Title/Abstract])))

AND ((y_5[Filter]) AND (english[Filter]))
